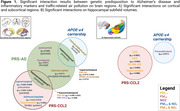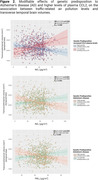# Genetic predisposition to AD and inflammation determine vulnerable brain regions to air pollution

**DOI:** 10.1002/alz.092103

**Published:** 2025-01-09

**Authors:** Natalia Vilor‐Tejedor, Patricia Genius, Blanca Rodríguez‐Fernández, Federica Anastasi, Marta Cirach, Mark Nieuwenhuijsen, Carolina Minguillón, Manel Esteller, Arcadi Navarro, Juan Domingo Gispert

**Affiliations:** ^1^ Barcelonaβeta Brain Research Center (BBRC), Pasqual Maragall Foundation, Barcelona Spain; ^2^ Hospital del Mar Research Institute (IMIM), Barcelona Spain; ^3^ Department of Genetics, Radboud Medical University Center, Nijmegen Netherlands; ^4^ Centre for Genomic Regulation (CRG), Barcelona Institute of Science and Technology (BIST), Barcelona Spain; ^5^ ISGlobal, Barcelona Institute for Global Health ‐ Campus MAR, Barcelona Biomedical Research Park, Barcelona Spain; ^6^ Centro de Investigación Biomédica en Red de Epidemiología y Salud Pública (CIBERESP), Madrid Spain; ^7^ Barcelonaβeta Brain Research Center (BBRC), Barcelona Spain; ^8^ Centro de Investigación Biomédica en Red de Fragilidad y Envejecimiento Saludable (CIBERFES), Instituto de Salud Carlos III, Madrid Spain; ^9^ Josep Carreras Leukaemia Research Institute (IJC), Badalona, Barcelona Spain; ^10^ Institució Catalana de Recerca i Estudis Avançats (ICREA), Barcelona Spain; ^11^ Physiological Sciences Department, School of Medicine and Health Sciences, University of Barcelona (UB), Barcelona, Catalonia Spain; ^12^ Centro de Investigación Biomédica en Red Cancer (CIBERONC), Madrid Spain; ^13^ Institute of Evolutionary Biology (CSIC‐UPF) Universitat Pompeu Fabra, Barcelona Spain; ^14^ Centro Nacional de Investigaciones Cardiovasculares (CNIC), Madrid Spain; ^15^ Centro de Investigación Biomédica en Red Bioingeniería, Biomateriales y Nanomedicina, Madrid Spain

## Abstract

**Background:**

The detrimental effects of air pollution on health are well‐documented, yet its impact on brain structure in the early asymptomatic stages of Alzheimer’s disease (AD) remains under‐explored. This study investigated the relationship between air pollution and brain imaging features, focusing on the moderating role of genetic factors associated with AD and inflammation.

**Methods:**

A total of 1,153 individuals from the ALFA cohort, many within the Alzheimer’s continuum, with available genotyping, air pollution estimation and magnetic resonance imaging were included (62.6% women, average age 55,6 years). Land use regression models were used to estimate individual levels of air pollution, including nitrogen oxide (NO_2_) and particulate matter (PM_2.5_, PM_10_), at the participants’ residential addresses. Functional genetic scores, including plasma pQTLs for inflammatory markers (PRS‐CCL2, PRS‐IL6, PRS‐CCL19), CSF pQTLs for AD pathology (PRS‐Aβ42, PRS‐ptau), genetic predisposition to clinical AD (PRS‐AD), and APOE‐ε4 carriership, were assessed. General linear models, corrected for age at MRI visit, sex and years of education, were computed to assess the main effects of air pollution on brain volumes. Modifiable genetic effects in gene‐environment interaction models were investigated.

**Results:**

Individuals at higher genetic predisposition to AD and APOE‐ε4 carriers exhibited increased vulnerability to air pollution, particularly in temporal and frontal regions [Figure 1A]. Individuals with high genetic predisposition for inflammatory markers showed distinct patterns of vulnerability, particularly affecting regions within the parietal and limbic lobes, with notable impact on hippocampal volumes and the subfields within the hippocampal formation [Figure 1B]. Finally, as pollution levels increased, reduction in transverse temporal volumes was observed in individuals at high risk of AD and altered CCL2 protein plasma levels [Figure 2]. Multiple comparison corrections were performed within brain lobes by regions.

**Conclusions:**

Our results suggested a differential impact of air pollution on brain structure, modulated by genetic predisposition to AD and inflammation. This underscores the importance of considering genetic information when assessing the neurological impact of environmental exposures. These findings pave the way for more personalized approaches in public health interventions, and highlight the need for targeted strategies to mitigate the impact of environmental factors on neurological health.